# Anchorless risk or released benefit? An updated view on the ADAM10-mediated shedding of the prion protein

**DOI:** 10.1007/s00441-022-03582-4

**Published:** 2022-01-27

**Authors:** Behnam Mohammadi, Feizhi Song, Andreu Matamoros-Angles, Mohsin Shafiq, Markus Damme, Berta Puig, Markus Glatzel, Hermann Clemens Altmeppen

**Affiliations:** 1 Institute of Neuropathology, University Medical Center Hamburg-Eppendorf (UKE), Hamburg, Germany; 2 Institute of Biochemistry, Christian-Albrechts-University Kiel, Kiel, Germany; 3 Department of Neurology, Experimental Research in Stroke and Inflammation (ERSI), University Medical Center Hamburg-Eppendorf (UKE), Hamburg, Germany; 4 Working Group for Interdisciplinary Neurobiology and Immunology (INI Research), Hamburg, Germany

**Keywords:** Biomarker, Immune responses, Intercellular communication, Neurodegeneration, Proteolytic cleavage

## Abstract

The prion protein (PrP) is a broadly expressed glycoprotein linked with a multitude of (suggested) biological and pathological implications. Some of these roles seem to be due to constitutively generated proteolytic fragments of the protein. Among them is a soluble PrP form, which is released from the surface of neurons and other cell types by action of the metalloprotease ADAM10 in a process termed ‘shedding’. The latter aspect is the focus of this review, which aims to provide a comprehensive overview on (i) the relevance of proteolytic processing in regulating cellular PrP functions, (ii) currently described involvement of shed PrP in neurodegenerative diseases (including prion diseases and Alzheimer’s disease), (iii) shed PrP’s expected roles in intercellular communication in many more (patho)physiological conditions (such as stroke, cancer or immune responses), (iv) and the need for improved research tools in respective (future) studies. Deeper mechanistic insight into roles played by PrP shedding and its resulting fragment may pave the way for improved diagnostics and future therapeutic approaches in diseases of the brain and beyond.

## Introducing the proteolytic processing of the prion protein: a brief overview on a protein’s enzymatic fragmentation

Since its discovery in the context of unravelling a mysterious group of fatal and transmissible neurodegenerative disease in humans and animals (now collectively termed prion diseases) (Colby and Prusiner 2011; Prusiner 1993), a variety of biological functions and diverse (patho)physiological implications (reviewed in (Aguzzi et al. 2008; Hirsch et al. 2017; Manni et al. 2020; Watts et al. 2018; Wulf et al. 2017)) have been attributed to the evolutionary conserved cellular prion protein (PrP) (Basler et al. 1986; Oesch et al. 1985; Westaway and Prusiner 1986). In prion diseases (including Creutzfeldt-Jakob disease [CJD] in humans or bovine spongiform encephalopathy [BSE] in cattle), PrP undergoes progressive, templated three-dimensional misfolding (into its pathological ‘scrapie’ isoform PrP^Sc^) and aggregation, and its expression is thus prerequisite and driving force of these ultimately fatal neurodegenerative conditions (Bockman et al. 1985; Prusiner 1982). Another pathological implication was found roughly a decade ago, when it was first shown (Gimbel et al. 2010; Laurén et al. 2009) and subsequently firmly established (Beraldo et al. 2016; Chen et al. 2010; Chung et al. 2010; Dohler et al. 2014; Freir et al. 2011; Gomes et al. 2019; Hu et al. 2014; Klyubin et al. 2014; Larson et al. 2012; Nicoll et al. 2013; Resenberger et al. 2011; Salazar et al. 2017; Um et al. 2013, 2012) that harmful protein conformers associated with more common neurodegenerative proteinopathies bind to PrP at the neuronal cell surface and thereby initiate neurotoxic signalling cascades. To date, this detrimental interaction with PrP has been shown for oligomers of Amyloid-β (Aβ), tau, and α-synuclein, which are critically associated with Alzheimer’s disease (AD), frontotemporal dementia and other tauopathies, or Parkinson’s disease, respectively (Corbett et al. 2020; Ferreira et al. 2017; Hu et al. 2018; Ondrejcak et al. 2018).

But what about the multitude of suggested physiological functions? A rather small glycoprotein at the cell surface acting like a ‘Jack-of-all-trades’? Even though some suggested roles are clearly controversial, have been challenged, or did not withstand experimental verification (e.g., upon developing improved knockout mice lacking genetic confounding effects (Nuvolone et al. 2016)), PrP^C^ most certainly can be regarded as a ‘multifunctional protein’. However, this multifunctional character might not solely be restricted and immanent to the — so far — primarily studied mature, membrane-anchored and full-length form of PrP (fl-PrP). Likewise, it might not be limited to the nervous system, the area where most prion research of the past has focused on. Increasing evidence reveals that both, transient functional interactions with diverse binding partners (Aguzzi et al. 2008; Béland and Roucou 2012; Linden 2017) and endogenously produced forms or fragments of PrP holding intrinsic biological properties (Collins et al. 2018; Guillot-Sestier and Checler 2012; Linsenmeier et al. 2017), critically contribute to the protein’s apparent versatility. The latter aspect of enzymatically generated fragments constitutes the focus of this review. In particular, the release of nearly full-length PrP upon membrane-proximate cleavage by a metalloprotease, the so-called ‘shedding’ event, and current knowledge and perspectives in that regard will be discussed in detail. For the sake of completeness, however, we will start with a brief introduction of additional cleavage events occurring on PrP and their relevance in physiological and/or pathological conditions. For a more comprehensive view of these cleavages, we refer to earlier review articles (Altmeppen et al. 2013, 2012; Dexter and Kong 2021a,  b; Liang and Kong 2012; Linsenmeier et al. 2017).

A process termed α-cleavage in the middle of the protein sequence separates the two structurally different parts of PrP (Chen et al. 1995; Haigh et al. 2009b; Harris et al. 1993; Linsenmeier et al. 2017): The intrinsically disordered N-terminal half or ‘flexible tail’, an important hub for interactions with diverse physiological and pathological ligands within fl-PrP (Béland and Roucou 2012; Carulla et al. 2015; Resenberger et al. 2011; Trevitt et al. 2014; Turnbaugh et al. 2012, 2011), is released into the extracellular space as a soluble (and rather instable (Mohammadi et al. 2020)) N1 fragment. N1 is a ligand linked with (neuro)protective and apparently myelin-maintaining activities as well as regulatory roles in diverse cellular processes and cell-to-cell communication (Carroll et al. 2020; Collins et al. 2018; Guillot-Sestier et al. 2012, 2009; Küffer et al. 2016; Mohammadi et al. 2021, 2020). The counterpart of N1, a globularly structured, N-glycosylated and stable C1 fragment, remains attached to the cell surface via PrP’s C-terminal GPI-anchor (Chen et al. 1995; Harris et al. 1993; Shyng et al. 1993). Upon α-cleavage, PrP’s central hydrophobic domain gets exposed as C1’s new N-terminus, which may have functional consequences, for instance, in cell-to-cell interactions or binding of certain ligands (Altmeppen et al. 2012; Bremer et al. 2010; Brenna et al. 2020; Harris et al. 1993; Linsenmeier et al. 2017). Since the N-terminal tail is critical for binding toxic protein assemblies mentioned above, α-cleavage can be regarded as a protective event rendering PrP unresponsive to these conformers. Moreover, the C1 fragment is resistant to misfolding in prion diseases and can even impair this process (Lewis et al. 2009; Westergard et al. 2011). Although α-cleavage represents the major physiological cleavage event of PrP in many cell types and its resulting fragments may hold relevant functions, there is enduring controversy on the responsible protease(s) (Altmeppen et al. 2011; Béland et al. 2012; Haigh et al. 2009b; Laffont-Proust et al. 2005; Liang et al. 2012; Mays et al. 2014; McDonald et al. 2014; Oliveira-Martins et al. 2010; Pietri et al. 2013; Taylor et al. 2009; Vincent et al. 2001; Wik et al. 2012), and it may well be that more than just one proteolytic entity ensures this important cleavage. Independent of the identification of the relevant protease(s), recent reports have shown that dimerization of PrP (Béland et al. 2012) or binding of PrP-directed peptide aptamers (Corda et al. 2018) causes increased α-cleavage, which may hold therapeutic relevance.

Around amino acid 90 and, thus, slightly N-terminally shifted from the α-cleavage site, the so-called β-cleavage by proteases or reactive oxygen species (with the latter causing a Fenton reaction) may occur, which is increased under pathological conditions and/or in response to oxidative stress (Castle et al. 2019; Chen et al. 1995; Mangé et al. 2004; Mays et al. 2014; McMahon et al. 2001). In consequence of this cleavage, a shorter N2 fragment is released while a longer C2 fragment stays attached to the membrane. Both fragments have been suggested to hold intrinsic functions and pathological roles (Haigh et al. 2015, 2009a, b; Lau et al. 2015; Sunyach et al. 2007).

The γ-cleavage represents the most recently described cleavage event (Lewis et al. 2016), preferentially occurs on unglycosylated PrP and separates a long N-terminal (N3 of ~ 20 kDa) from a short C-terminal fragment (C3 of ~ 6 kDa). Knowledge of its (patho)physiological implications is limited, yet it appears to be upregulated in prion disease.

Additional proteolytic fragmentation of synthetic PrP peptides by certain metalloproteases has been shown in vitro (Kojima et al. 2014), yet whether all cleavages found with recombinant protease-substrate mixtures in the test tube also hold in vivo-relevance is currently unsolved (Linsenmeier et al. 2017; McDonald et al. 2014).

Following this overview, we will now concentrate on another relevant cleavage event, termed shedding, occurring in the far C-terminal part of PrP and increasingly raising scientific attention.

## Difficulties detecting shed PrP: new research tools enabling novel insights

**Fig. 1:**
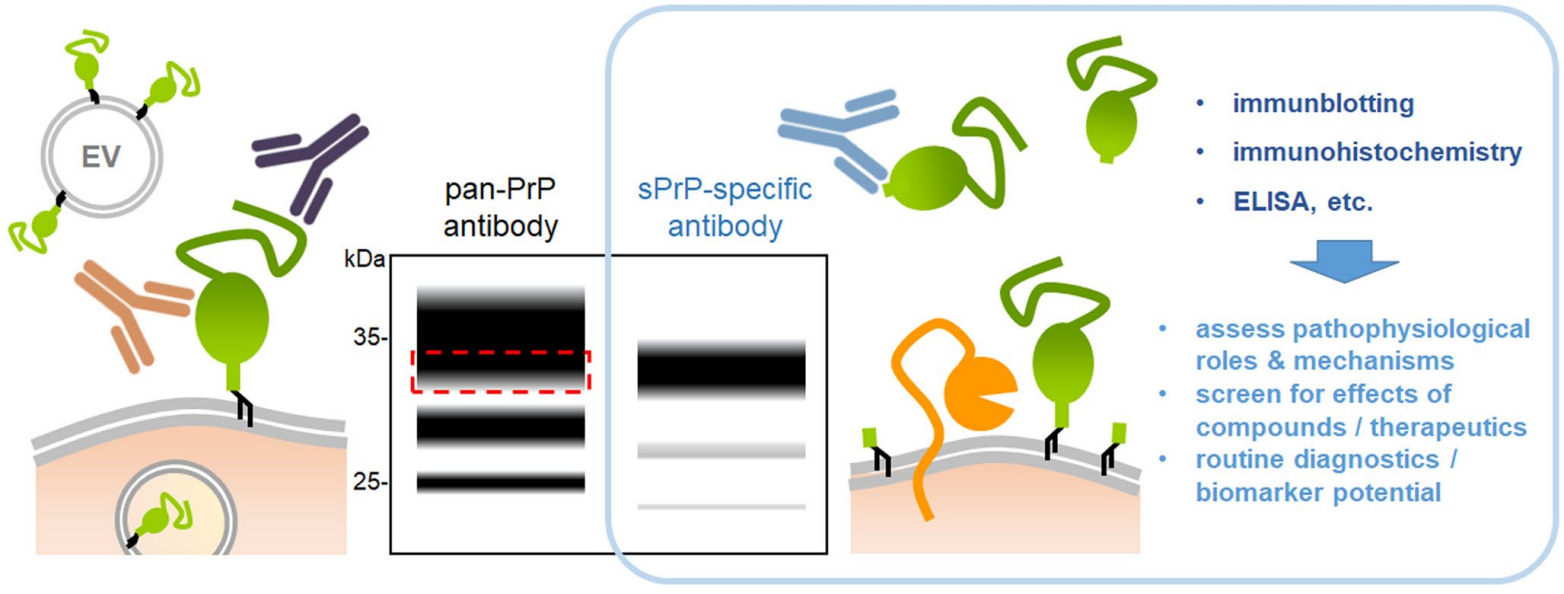
Challenging assessment of sPrP and the advantage of site-specific antibodies. Besides membrane-attached forms of PrP (green) in/on cells or on extracellular vesicles (EV), plenty of different cleaved fragments (not all depicted here) are present in biological samples. Due to the similar size of sPrP (released by ADAM10; orange) and fl-PrP and the usually vast excess of the latter, pan-PrP antibodies do not discriminate between these forms (e.g., in immunoblots), and sPrP is therefore masked (as indicated by the schematic immunoblot in the middle (red striped box). The three bands typical for PrP are caused by its glycosylation state (with di-, mono- , and unglycosylated forms; N-glycans are not depicted here to simplify matters). Generation of cleavage site-specific antibodies (blue) allows for reliable detection of sPrP (right lane in the blot; note the slightly lower molecular weight due to the lack of the GPI-anchor, and the strong predominance of diglycosylated sPrP (Linsenmeier et al. 2018)). Such fragment-specific antibodies allow for a reliable assessment of specific PrP derivatives (in this case sPrP) in standard research and routine diagnostic methods, such as western blot, histological approaches, or ELISA. This enables studies on the (patho)physiological relevance as well as on the therapeutic and/or diagnostic potential of certain PrP fragments

The presence of extracellular PrP molecules with nearly full-length protein sequence has been described roughly three decades ago (Borchelt et al. 1993; Harris et al. 1993; Tagliavini et al. 1992), and although their physiological production by the endogenous metalloproteinase ADAM10 (in a process referred to as ‘proteolytic shedding’) is known for more than a decade by now (Altmeppen et al. 2011; Taylor et al. 2009), only little insight into the biological relevance of this shed PrP (sPrP) has been gained until recently. Difficulties in reliable identification of sPrP in experimental models and complex biological tissues certainly represent the main reason for this lack of knowledge. In contrast to truncated released or cell-associated fragments resulting from the α- or β-cleavage, which differ remarkably from fl-PrP in size/molecular weight and may be easily discriminated by western blot analyses of conditioned media/body fluids or cell/tissue lysates, respectively, sPrP is usually masked by levels of fl-PrP present in vast excess and, thus, simply ‘overlooked’ when using common (pan-) PrP antibodies for detection (Fig. 1). To solve this problem, we have recently generated a cleavage-site specific antibody for sensitive and reliable detection of sPrP (Linsenmeier et al. 2018), based on sequence and cleavage-site information for murine PrP published earlier (Taylor et al. 2009). The glycosylation state of PrP critically influences PrP’s biology and pathophysiological roles (DeArmond et al. 1997; Makarava et al. 2020; Nishina et al. 2006; Priola and Lawson 2001; Puig et al. 2011). Our new sPrP antibody revealed that PrP is mostly shed in a diglycosylated state, which likely represents the physiological status-quo at the cell surface, where shedding by ADAM10 is thought to occur. Moreover, we have subsequently identified the shedding process as a relevant part of a posttranslationally active regulatory network controlling cellular PrP homeostasis (Linsenmeier et al. 2018). This compensatory network also involves cellular uptake and degradation, as well as release of PrP via extracellular vesicles (Brenna et al. 2020; D’Arrigo et al. 2021; Falker et al. 2016; Fevrier et al. 2004; Guo et al. 2016; Ritchie et al. 2013; Wik et al. 2012). Hence, PrP release via EVs (Heisler et al. 2018) or the ADAM10-mediated shedding are increased upon lysosomal inhibition (Linsenmeier et al. 2018) and in mice lacking intracellular regulators of retrograde sorting and vesicular transport to lysosomes (Heisler et al. 2018; Linsenmeier et al. 2018; Uchiyama et al. 2017). Vice versa, pharmacological inhibition of proteolytic PrP shedding is compensated by an elevated release of EVs with increased PrP load (Linsenmeier et al. 2018). We also found evidence for ‘cleavage cascades’ occurring on PrP, as the truncated C1 fragment resulting from the α-cleavage can subsequently be shed by ADAM10 and thus be detected with a sPrP-specific antibody, for instance, in conditioned media (Linsenmeier et al. 2018). The same seems plausible for the β-cleavage product C2 (Perini et al. 1996). This further complicates the variety of released PrP fragments and investigations on their particular functions or pathological relevance. Therefore, improved assays, such as capillary western analysis (Castle et al. 2019), in combination with powerful site-specific antibodies will lead to better discrimination, reliable quantifications, and eventually biological insight (Fig. 1). For sPrP, this has already been achieved in parts and will certainly continue to reveal novel (patho)physiological implications, as discussed in the following paragraphs.

## Biological roles and assessment of sPrP in neurodegenerative proteinopathies

### Prion diseases

**Fig. 2:**
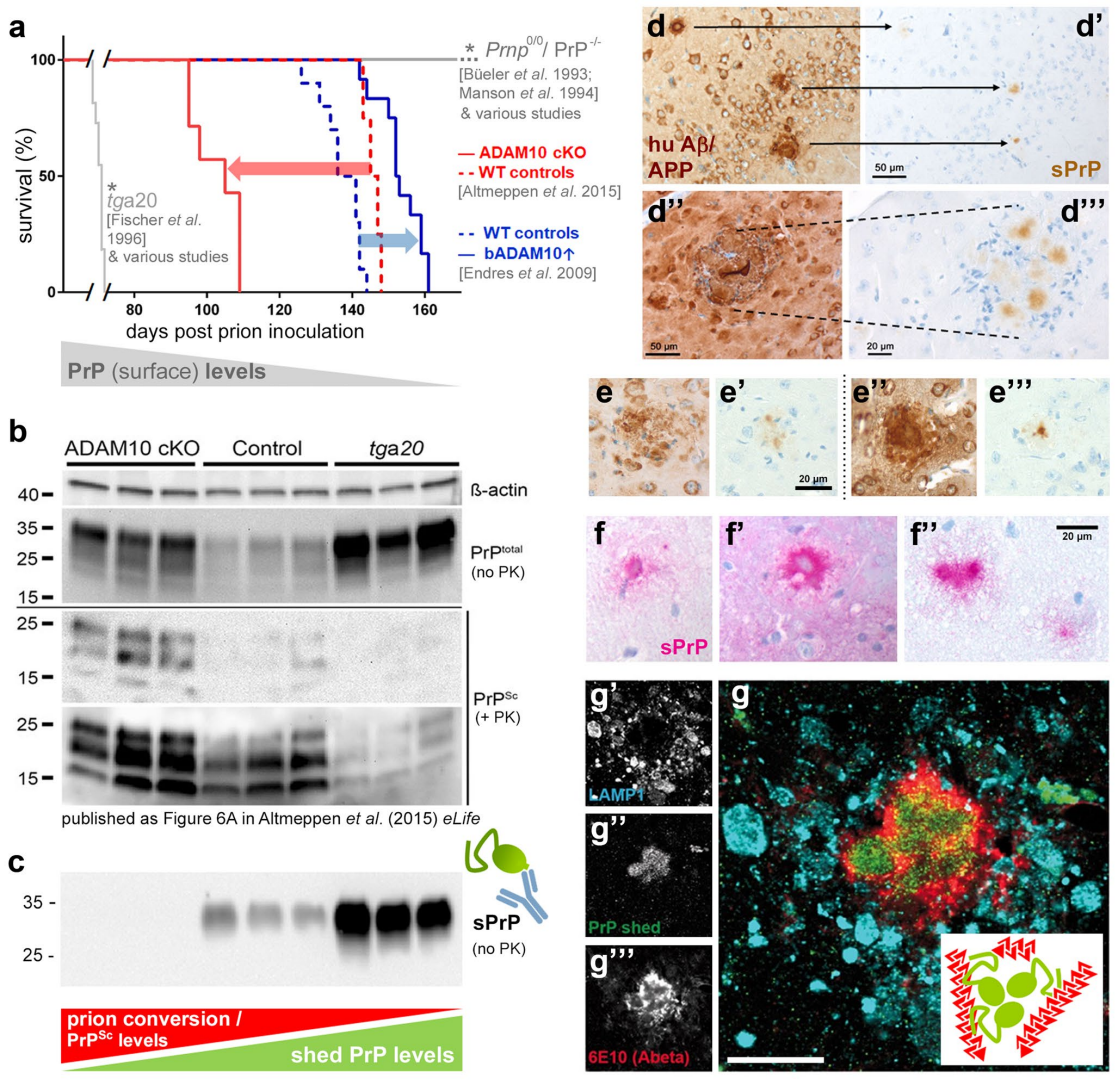
Consequences of the ADAM10-mediated shedding of PrP in neurodegenerative diseases. (**a**) Kaplan–Meier survival curves summarizing two in vivo studies that assessed the role of ADAM10 in prion diseases (with both studies using the Rocky Mountain Laboratory (RML) prion strain). While moderate overexpression of bovine ADAM10 in mice (bADAM10↑; blue line) in the study of Endres et al. (2009) resulted in prolonged survival (blue arrow; wild-type controls represented by dotted blue line), lack of ADAM10 in forebrain neurons (in ADAM10 cKO mice; red line) caused shortened incubation times (red arrow) compared to controls (dotted red line) (Altmeppen et al. 2015). For comparison, the diagram also schematically presents prion protein knockout mice (e.g., *Prnp*^0/0^ [(Büeler et al. 1993)] or PrP^−/−^ [(Manson et al. 1994)]), which are resistant to prion infection, as well as PrP-overexpressing mice (e.g., tga20 [(Fischer et al. 1996)]), which succumb to disease very early. *Note that the curves for these models reflect typical study outcome rather than exact datasets. Taken together, all models/genotypes depicted here fit the view that levels of (cell-associated) PrP^C^ critically determine survival times in prion diseases (Manson et al. 1994; Sandberg et al. 2011) (see grey ‘correlation bar’ below). (**b**) Western blot data reproduced from Fig. 6A in Altmeppen et al. (2015) *eLife* ((Altmeppen et al. 2015); https://elifesciences.org/articles/04260) published under a CC BY 4.0 license (https://creativecommons.org/licenses/by/4.0/). In non-PK digested samples, highest levels in total PrP (i.e., PrP^C^ and PrP^Sc^) were found in terminally diseased tga20 mice (at 65 days post-inoculation, dpi), followed by ADAM10 cKO and wild-type control mice (both at 95 dpi). In contrast, prion conversion (judged by PrP^Sc^ amounts detectable after PK digestion) was highest in ADAM10 cKO while barely detectable in tga20 mouse brains. (**c**) Re-analysis of aforementioned samples in a replica blot probed with a new sPrP-specific antibody demonstrates lack of detectable sPrP in ADAM10 cKO and efficient sPrP production in tga20 mice. In connection with (**b**) this may indicate an inverse correlation (see red/green ‘correlation bars’) between PrP shedding and pathogenic prion conversion (though deeper insight is clearly required). (**d,** **d″**) Amyloid plaques composed of human Aβ (left) in brain sections of 8 months-old APP23 mice heterozygous for PrP (*Prnp*^ + / −^). Note that endogenous sPrP (stained for in serial sections shown on the right; **d′**, **d‴**) is enriched in the plaques (showing multiple foci in the lower panel; **d‴**). (**e**, **e′**, **e″**, **e‴**) In APP23 mice with normal PrP expression (*Prnp* ^+ / +^) colocalization of sPrP with diffuse (left panel) and dense amyloid plaques (right panel) is already detectable at 5 months of age. (**f**, **f′**, **f″**) Plaque-like appearance of sPrP (detected with alkaline phosphatase, hence the pink signal) in brain sections of another AD mouse model (5xFAD mouse, 5 months old). (**g**) Immunofluorescence analysis showing a brain section of a 5xFAD mouse stained for Aβ plaques (red), sPrP (green), and endosomal/lysosomal marker LAMP1 (bright blue; mostly representing dystrophic neurites around plaques). Note that sPrP is enriched in the centre of this amyloid plaque (merged channels; **g**). Scale bar in the magnified merge picture is 30 µm. Inlay shows a model of the conceivable Aβ (red) sequestrating and plaque-promoting action of sPrP (green). Respective non-coloured single channels are shown on the left (**g′**, **g″**, **g‴**)

In stark contrast to the aforementioned harmful roles in neurodegenerative diseases played by fl-PrP at the cell surface, diffusible extracellular forms or derivatives of PrP have been shown to protect from prion misfolding and act against toxic protein assemblies. Expression of a soluble dimerized PrP in prion-infected mice interfered with PrP^Sc^ formation and disease progression (Meier et al. 2003). Similar ‘anti-prion’ effects were also observed for recombinant PrP (Priola et al. 1994; Yuan et al. 2013) and for PrP being released upon treatment with lipid-raft disturbing drugs (Bate et al. 2009; Marella et al. 2002; Taraboulos et al. 1995) or overexpression of a sorting factor (SNX33) in prion-infected cell cultures (Heiseke et al. 2008). In the latter study, release of PrP was likely accomplished by phospholipases cleaving within the GPI-anchor structure (Caughey et al. 1989; Harris et al. 1993; Stahl et al. 1987). Soon after that study, the zinc-dependent metalloprotease ADAM10 was shown to mediate the proteolytic shedding of PrP just a few amino acids away from the GPI-anchor in vitro (Taylor et al. 2009). Subsequent studies in transgenic mice not only confirmed ADAM10 as the major PrP sheddase in vivo (Altmeppen et al. 2011), but also revealed its protective and disease-modifying effects upon prion infection (Altmeppen et al. 2015; Endres et al. 2009) (Fig. 2a). Until now, ADAM10 even seems to be the only relevant sheddase of PrP (Altmeppen et al. 2011; Linsenmeier et al. 2018; McDonald et al. 2014; Taylor et al. 2009). Shed PrP, the physiological correlate of soluble PrP forms mentioned above, seems to bind and block critical PrP^Sc^ assemblies (‘seeds’) in the extracellular space and thereby interfere with the conversion process, as indicated by an inverse correlation of sPrP and PrP^Sc^ levels shown in a recent study (Linsenmeier et al. 2021) and reflected in Fig. 2b, c. In addition to this blocking effect, sPrP may also act as a ligand (similar to PrP’s N1 fragment) inducing neuroprotective signalling cascades or rescuing PrP functions in transgenic mice expressing toxic PrP mutants (Race et al. 2009). Accordingly, lack of protective sPrP in transgenic mice expressing PrP with a C-terminal deletion (Δ214-229 (Puig et al. 2016)) and reduced PrP shedding in cells and mice expressing PrP with an altered GPI-anchor and, hence, shifted membrane localization (Puig et al. 2019, 2011) may contribute to the respective pathological phenotypes observed in these models.

**Fig. 3:**
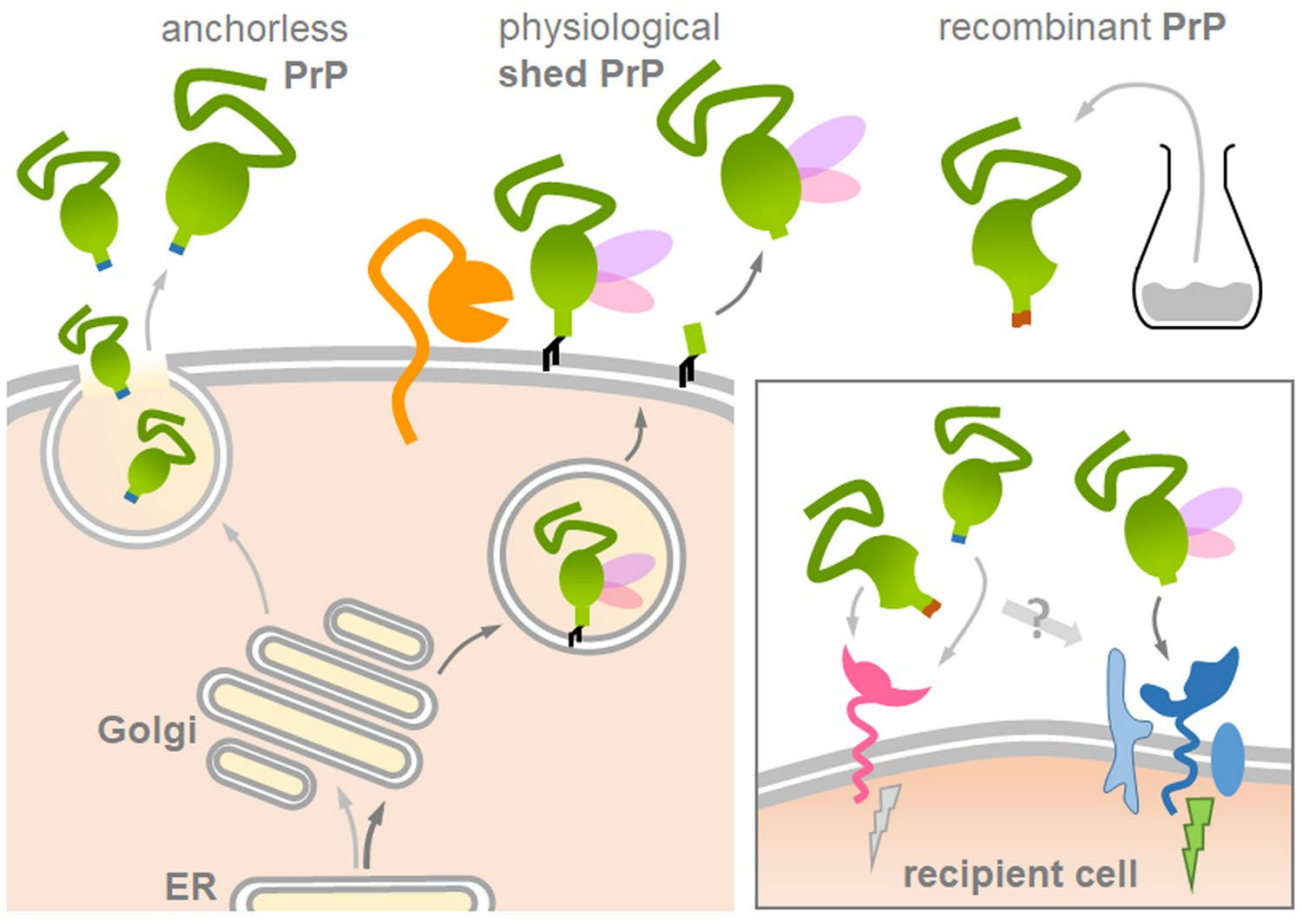
Structural differences between experimentally employed ‘anchorless’ PrP or recombinant PrP and physiological sPrP may affect biological functions and study outcome. Several studies on diverse topics covered in this review used transgenically expressed anchorless PrP (on the left) or recombinant PrP (on the upper right) as soluble PrP forms in assumed analogy to physiologically generated sPrP (middle). However, sPrP likely differs from these forms: recPrP is unglycosylated and anchorless PrP typically underglycosylated (no or one N-glycan), whereas sPrP is mainly diglycosylated (pink/purple structures). Moreover, the C-terminus is altered and the overall structure may be different. In sum, these features could feasibly affect critical ligand-to-receptor interactions and downstream effects (box on the lower right) and other biological implications. This should be considered and controlled for in experimental paradigms

A (seemingly) opposing finding, however, challenges the view of soluble PrP forms being protective: Anchorless PrP expressed in transgenic mice is a potent substrate for prion conversion and aggregate formation (Chesebro et al. 2010, 2005; Rangel et al. 2013; Rogers et al. 1993; Stöhr et al. 2011). Anchorless forms of PrP are also associated with some genetic forms of prion diseases in humans (Choi et al. 2016; Jansen et al. 2010; Zanusso et al. 2014). However, a key difference that may resolve this contradiction is the different N-glycosylation pattern between transgenically expressed or mutation-derived anchorless PrP on the one hand, and physiological sPrP on the other hand (Fig. 3): while the latter is predominantly diglycosylated (due to its transport through the secretory pathway as a GPI-anchored protein and subsequent cleavage after reaching the cell surface (Linsenmeier et al. 2018)), anchorless PrP is secreted in an underglycosylated state. This may have a profound impact on how these PrP forms encounter and affect extracellular PrP^Sc^ assemblies. In fact, an influence of the N-glycans on PrP’s susceptibility to prion conversion (depending on the respective prion strains) has been firmly established (DeArmond et al. 1997; Makarava et al. 2020; Nishina et al. 2006; Priola and Lawson 2001; Sevillano et al. 2020). In many experimental paradigms, underglycosylated PrP was efficiently converted, whereas diglycosylated PrP was a rather poor substrate for the templated misfolding (Camacho et al. 2019; Cheng et al. 2017; DeMarco and Daggett 2009; Kang et al. 2020; Priola and Lawson 2001; Xiao et al. 2013). This may well translate into sPrP’s blocking activity against the amplification of certain prion strains.

The situation, however, might be more complicated, as one in vitro study showed that ADAM10, in principal, is also able to shed misfolded PrP^Sc^ (Taylor et al. 2009). This could -to some degree- contribute to the spread of anchorless PrP^Sc^ assemblies and pathology within the brain (Altmeppen et al. 2015). Surprisingly, in contrast to the peptide bond hydrolysis by ADAM10, experimentally applied phospholipase C is incapable of cleaving the GPI-anchor of PrP^Sc^ (Caughey et al. 1990; Stahl et al. 1990). It remains to be studied whether ‘proteolytic shedding’ by ADAM10 and release of putative shed PrP^Sc^ seeds into body fluids also plays a role in the ‘environmental shedding’ of prions resulting in the high contagiosity observed in chronic wasting disease (CWD), a prion disease affecting deer, moose, and elk (Bessen et al. 2010; Denkers et al. 2020; Moore et al. 2016; Moreno and Telling 2018; Tennant et al. 2020). Expression of PrP with a single amino acid variation (found in cervid PrP) in mice affected prion strain selection upon infection with CWD prions (Bian et al. 2021). Though not investigated in that study, it would be interesting to assess whether this variation in close proximity to the cleavage site and GPI-anchor affects the shedding, with possible consequences for strain and disease features. Notably, a recent study investigating the role of the extracellular matrix component heparan sulfate as a cofactor in prion diseases revealed that prion deposits in brains of transgenic mice infected with CWD prions largely consisted of heparan sulfate-associated ADAM10-cleaved PrP^Sc^ (Aguilar-Calvo et al. 2020). Subsequent studies, however, provided evidence that ADAM10-cleaved PrP^Sc^ was mostly associated with large perivascular and possibly inert plaques, whereas diffusible oligomeric or sub-fibrillar (and presumably more neurotoxic) PrP^Sc^ assemblies dominating in many prion diseases mostly result from the conversion of GPI-anchored PrP (Aguilar-Calvo et al. 2020; Callender et al. 2020; Sevillano et al. 2020). In conclusion, the ADAM10-mediated shedding might play a dual role in prion diseases, and whether it is protective or rather disease-supporting might depend on critical molecular stoichiometries, cofactors, and currently unknown cellular modalities as well as species and prion strains. Regarding PrP^Sc^ formation and survival times of prion-infected mice, recent studies suggest that protective effects may dominate (Altmeppen et al. 2015; Endres et al. 2009; Linsenmeier et al. 2021).

### Alzheimer’s disease and other neurodegenerative proteinopathies

Considering cell surface PrP’s role as a receptor and toxicity mediator of harmful protein assemblies (introduced in paragraph 1), it is not surprising that released forms of PrP harbouring the relevant binding sites (Chen et al. 2010; Laurén et al. 2009) instead have the ability to bind and sequester oligomeric assemblies in the extracellular space and interfere with their neurotoxicity in respective model systems. This has most convincingly been shown for Aβ oligomers and their ‘neutralization’ by the N1 fragment resulting from the α-cleavage (Béland et al. 2014; Fluharty et al. 2013; Guillot-Sestier et al. 2012; Nieznanski et al. 2012; Resenberger et al. 2011; Scott-McKean et al. 2016). Several studies also employed anchorless or recombinant PrP (mimicking physiological sPrP) and found similar binding affinities for and protective effects against Aβ oligomers (Calella et al. 2010; Fluharty et al. 2013; König et al. 2021; Nieznanski et al. 2012; Scott-McKean et al. 2016). In fact, an additional binding site targeting the ends of Aβ fibrils has recently been located in PrP’s C-terminal half, which is therefore preserved in sPrP yet absent in N1 (Amin and Harris 2021; Bove-Fenderson et al. 2017). Some reports also indicated increased α-cleavage and shedding of PrP in AD models and brains, possibly reflecting a protective feedback loop (Béland et al. 2014; Ostapchenko et al. 2013). Similar to prion diseases discussed above, existing data for Aβ (possibly holding true for other harmful conformers alike) supports the view of a two-level protection conferred by the ADAM10-mediated shedding: First, this process reduces amounts of PrP as the toxicity receptor at the neuronal surface (Jarosz-Griffiths et al. 2019). Second, sPrP blocks toxic conformers and may support their sequestration into less toxic plaques. In fact, a plaque-promoting effect and presence of PrP in amyloid deposits have been demonstrated earlier (Boon et al. 2020; Ferrer et al. 2001; Schwarze-Eicker et al. 2005; Takahashi et al. 2021). These studies, however, used pan-PrP antibodies for detection. Most recently, using our site-specific antibody, we provided further insight that sPrP is enriched in the centre of amyloid plaques in mouse models for AD-associated amyloidosis (Linsenmeier et al. 2021) (Fig. 2d–g). Although doubtlessly many proteins are found (enriched) in plaques (among them rather specific interactors of Aβ or its precursor protein (APP), but also others just being trapped by these ‘sticky’ extracellular structures), many of the aforementioned studies speak in favour for sPrP playing an Aβ-sequestrating and plaque-promoting role. Given that large deposits, such as plaques, are currently considered less harmful than diffusible, toxic oligomeric species of neurodegeneration-associated misfolded proteins, this would indicate a protective role of sPrP. However, deeper mechanistic insight is certainly required.

The ability of PrP to bind toxic conformers has also been linked with a role in mediating their cellular uptake (De Cecco et al. 2020; Foley et al. 2020; Legname and Scialò 2020). Though not studied to date, in that scenario, ADAM10-mediated shedding of surface PrP could regulate these phagocytic activities. Moreover, sPrP bound to harmful extracellular oligomers could represent a signal triggering the binding to a given cell surface receptor (with homophilic interactions with membrane-anchored PrP being one conceivable possibility) and subsequent uptake.

### Therapeutic and biomarker potential of sPrP in neurodegeneration

Based on the above, stimulation of the ADAM10-mediated PrP shedding could represent a therapeutic option in prion and other neurodegenerative diseases (Jarosz-Griffiths et al. 2019). ADAM10 is already pharmacologically targeted in certain skin disease treatments and, in a recent trial in AD patients, its activity has already been stimulated using the vitamin A analog acitretin (Endres et al. 2014). However, in view of the multitude of critical roles and substrates of this protease in the brain and throughout the body (mentioned in part in the following sections), unwanted side effects to be expected with this rather systematic treatment may pose major challenges (Kuhn et al. 2016; Saftig and Lichtenthaler 2015; Wetzel et al. 2017). We may now have overcome this hurdle by identifying a substrate-specific approach (Linsenmeier et al. 2021), in which binding of certain ligands (e.g., antibodies) to PrP causes increased sPrP levels in the absence of overt toxicity. This may — at least in part — explain the protective effects of PrP-directed antibodies found in diverse cellular and animal models of prion diseases and AD (Chung et al. 2010; Enari et al. 2001; Féraudet et al. 2005; Freir et al. 2011; Gilch et al. 2003; Heppner et al. 2001; Laurén et al. 2009; Peretz et al. 2001).

A promising therapeutic approach currently pursued against prion diseases (that could likewise be beneficial in other neurodegenerative diseases) is to reduce the overall expression of PrP via antisense oligonucleotides (Minikel et al. 2020; Raymond et al. 2019; Vallabh et al. 2019). A possible combination therapy, i.e., reducing total PrP expression and stimulating the release of remaining PrP molecules, may even increase the benefit while preserving putative physiological functions of soluble extracellular PrP fragments.

Both, verification and implementation of any PrP-modifying therapy in pre-clinical and clinical trials (Minikel et al. 2019) as well as urgently required improved (and earlier) diagnosis of specific neurodegenerative diseases, will critically depend on the assessment of reliable biomarkers. Detection of alterations in a defined subset of PrP molecules will most certainly be superior to detection of bulk PrP with its diverse forms and fragments found in biological samples (Vallabh et al. 2019). This highlights the relevance of fragment-specific antibodies and warrants the need for future studies on the potential of sPrP as a meaningful biomarker (Fig. 1) (Linsenmeier et al. 2018). A recent report of increased levels of ADAM10 in the brains of patients with CJD further supports this notion (Diaz-Lucena et al. 2021).

Although not directly assessed to date and therefore rather hypothesized below, it is conceivable that sPrP is mechanistically involved or could at least represent a relevant biomarker in several other pathological processes, as will be discussed in the next paragraph.

## Potential relevance of PrP shedding in other pathological processes

Both, the prion protein (Rubenstein et al. 2017; Sekar et al. 2019) and its sheddase ADAM10 (Appel et al. 2021; Sun et al. 2017; Warren et al. 2012; Zohar et al. 2011) have — so far independently — been implicated in pathological and recovery-associated processes following traumatic brain injury (TBI). Moreover, levels of soluble PrP were found elevated in blood plasma where they may serve as a diagnostic marker for TBI and sport-related concussion (Persad et al. 2021; Pham et al. 2015a,  b). Also, ADAM10 levels correlated with clinical grade (Persad et al. 2021). Although it seems likely that soluble PrP assessed in these studies correlates with sPrP, final proof needs to be obtained in systematic studies using specific antibodies to discriminate from other PrP fragments or from PrP released via extracellular vesicles. This will also help to investigate potential protective or regenerative processes conferred by sPrP. However, as inhibition of ADAM10 in a mouse model for TBI reduced tissue injury and inflammatory responses, it appears questionable if sPrP would act beneficially in this context (Appel et al. 2021).

PrP and its released fragments also seem to play beneficial roles in hypoxic conditions affecting the CNS, such as stroke (Doeppner et al. 2015; Guillot-Sestier et al. 2009; McLennan et al. 2004; Shyu et al. 2005; Spudich et al. 2005; Weise et al. 2006, 2004). While signalling and downstream effects mediated via cell surface PrP most certainly underlies some of the protective roles (reviewed in (Puig et al. 2020)), release of PrP fragments, such as sPrP and PrP, on extracellular vesicles may be relevant for intercellular communication with nearby or distant brain regions, neuron-glia interactions, or recruitment/activation of cell types required for the induction of regenerative processes, such as angiogenesis (Brenna et al. 2020; D’Arrigo et al. 2021; Guitart et al. 2016; Mitsios et al. 2007; Turu et al. 2008). Again, clarification of specific roles of sPrP in these aspects seems reasonable, as they could be employed therapeutically.

While all previously discussed (potential) roles of sPrP were exclusively focussed on the CNS, sPrP may reveal itself as a relevant molecule beyond this organ system. Moreover, in contrast to the aforementioned beneficial implications, sPrP may also carry out negative roles as discussed first for the aspect of tumorigenesis and cancer.

Why cancer? Increased PrP expression has been found in various types of malignant tumours ranging from brain tumours to breast, gastric, skin, and colorectal cancer. In these and other tumour entities, PrP was shown to support tumorigenesis and metastasis by engaging in a variety of pathogenic processes, including anti-apoptotic signalling cascades, cancer stem cell survival, angiogenesis, and even resistance towards chemotherapy and radiation (Atkinson et al. 2019; Barbieri et al. 2011; Bernardino-Sgherri et al. 2021; Corsaro et al. 2016; de Lacerda et al. 2016; Du et al. 2013; Ghazi et al. 2021; Le Corre et al. 2019; Li et al. 2009; Liang et al. 2007; Lopes et al. 2015; Luo et al. 2017; Pan et al. 2006; Roucou et al. 2005; Thellung et al. 2019; Wang et al. 2016; Yap and Say 2012). As such, elevated PrP levels may correlate with malignancy and are considered a sign for poor prognosis.

Strikingly, a huge amount of published evidence also implicates ADAM10 in various aspects of cancer development and progression. Previous studies mostly focussed on ADAM10’s role in extracellular matrix degradation for angiogenesis and metastasis or on its processing of cellular substrates regulating differentiation and cancer cell survival, yet did not consider or reveal any link to PrP (Crawford et al. 2009; Dempsey 2017; Ostalecki et al. 2017; Smith et al. 2020). However, this may now change given that a recent report found both, PrP and its sheddase, to be associated in the pathogenic process of breast cancer progression (Cheng et al. 2021). From the combination of elevated PrP levels and increased ADAM10 expression/activity found in various cancer types, one can anticipate that sPrP, the product likely generated by this molecular encounter, may be mechanistically involved in certain oncogenic processes. Considering the well-established role of PrP in signalling (Chiarini et al. 2002; Mattei et al. 2020; Mouillet-Richard et al. 2000) and the growing evidence for sPrP acting as a ligand or trophic factor in intercellular communication (discussed herein), further studies are warranted to check for a mechanistic relevance in cancer biology. But even if sPrP turns out to be not much more than a bystander, the combination of upregulated ADAM10 and PrP independently described in various cancer types and models could well point towards a diagnostic biomarker potential of sPrP assessable in body fluids.

Notably, two recent reports specifically linked released PrP with development of certain CNS tumours (Provenzano et al. 2017) and with chemotherapy resistance in breast cancer (Wiegmans et al. 2019). However, these studies did not strictly discriminate between PrP on extracellular vesicles or sPrP, which supports the need for further studies using differentiating antibodies and protocols.

In recent years, protective functions have also been attributed to PrP in the kidney (Han et al. 2020; Yoon et al. 2021; Zhang et al. 2015), and PrP is ‘secreted’ into the urine in response to chronic kidney disease and ER stress in kidney injury (Bignon et al. 2020). However, before assessment of PrP in plasma can be used as a reliable biomarker, differentiation between sPrP and other PrP forms again seems appropriate.

Lastly, shedding of PrP might be relevant in immune regulation, the immune privilege of certain organs, and inflammatory responses during (viral) infections. In fact, PrP is highly expressed in lymphoid tissues and has long been discussed to be important in the communication between immune cells (Bakkebø et al. 2015). It is tempting to speculate that sPrP (and other released PrP fragments) act similar to chemokines or interleukins and mediate intercellular crosstalk between diverse cell types as well as between different tissues and organ systems, such as neuro-immune interactions (Salvesen et al. 2019). These processes might be diverted and hence become harmful in tumorigenesis or CNS infections as mentioned earlier. Platelets (Perini et al. 1996), primary lymphoid cells (Parizek et al. 2001), and mast cells were shown to release PrP (Haddon et al. 2009), the latter especially upon activation, thus indicating functional relevance. It seems likely that this release is mediated by ADAM10 at the cell surface. After finding increased levels of soluble PrP in the CSF (Roberts et al. 2010) of patients with HIV-associated neurological impairment (Price et al. 1988) and suggesting soluble PrP as a respective biomarker (Megra et al. 2013), one group recently proposed a disease-accelerating role for shed PrP in HIV neuropathogenesis (Megra et al. 2017). In that scenario, active ADAM10 and PrP shedding are upregulated on astrocytes in response to certain inflammatory mediators. This, in turn, triggers a cascade of events eventually leading to increased monocyte recruitment to the brain and worsening of brain damage. Again, since pan-PrP antibodies were used in that study and no ultracentrifugation was performed (to exclude extracellular vesicles), a clear demonstration that bona-fide sPrP caused this effect is still pending.

## Further biological roles influenced by shedding or played by ‘sPrP’: a few facts and some fiction

An outstanding aspect common to many of the aforementioned putative implications of sPrP is its supposed role as a diffusible ligand in intercellular communication. In this regard, it appears likely that sPrP and/or the N1 fragment are the physiologically relevant ligands for a G-protein coupled receptor on Schwann cells, ensuring myelin maintenance in the peripheral nervous system (Henzi et al. 2020; Küffer et al. 2016).

Further support for the view that sPrP acts as a ligand in various processes also comes from several studies using recombinant PrP. Treatment of neurons with the latter, for instance, causes neuronal polarization, increased axon length and dendritic differentiation as well as synapse formation. Notably, C- or N-terminal PrP fragments were not sufficient to elicit this effect (Kanaani et al. 2005). Moreover, axons grow towards a source of recPrP, and cell surface PrP itself seems to act as ‘its own’ neuronal receptor in this process (Amin et al. 2016). This suggests that physiological sPrP may hold properties as both, a growth factor-like and chemoattractant molecule in neuronal differentiation, neuritogenesis, and synaptic homeostasis. Fittingly, ADAM10 holds key roles in brain development, axon targeting and functioning of synapses (Jorissen et al. 2010; Kuhn et al. 2016; Malinverno et al. 2010; Prox et al. 2013), although its substrate PrP was not specifically assessed in these studies.

One way by which PrP modulates signalling cascades is via interaction with different transmembrane partners. An inhibitory effect on excitotoxicity is mediated by binding of PrP to the NMDA receptor (Huang et al. 2021; Khosravani et al. 2008; Meneghetti et al. 2019; Petit-Paitel et al. 2012), whereas its interaction with this receptor and metabotropic glutamate receptor mGluR5 mediates toxicity upon binding of harmful protein oligomers (Hamilton et al. 2015; Hu et al. 2014; Resenberger et al. 2011; Um et al. 2013; You et al. 2012). Binding of PrP to the 37 kDa/67 kDa laminin receptor precursor (LRP/LR) (Gauczynski et al. 2001; Hundt et al. 2001; Simoneau et al. 2003) or the low-density lipoprotein receptor-related protein 1 (LRP1) (Parkyn et al. 2008; Taylor and Hooper 2007) (note the similar nomenclature!) regulates PrP’s cellular trafficking and homeostasis and may affect PrP-related signalling and prion conversion (Leucht et al. 2003; Mattei et al. 2020; Pinnock et al. 2016; Rushworth et al. 2013). Interaction of PrP with the neural cell adhesion protein (NCAM) at the cell surface is involved in cell adhesion and morphogenesis, neuronal differentiation, and neurite branching (Brethour et al. 2017; Prodromidou et al. 2014; Santuccione et al. 2005; Schmitt-Ulms et al. 2001; Slapšak et al. 2016). Like PrP and ADAM10 (discussed in Sect. 4), many of these PrP interactors are also associated with different processes during cancerogenesis (reviewed in (Colombo and Meldolesi 2015; Gonias and Campana 2014; Vania et al. 2019)). Since all relevant binding sites for interactions with these and other receptors are preserved in sPrP, this calls for detailed studies investigating whether sPrP may act as an antagonistic, agonistic or regulatory ligand of the diverse processes mentioned above. In fact, functional interaction with NCAM *in trans* was shown for recPrP (Chen et al. 2003; Santuccione et al. 2005). Intriguingly, a recent report demonstrated that experimentally administered recPrP induced MAP kinase Erk1/2 signalling via engagement of both, LRP1 and NMDA receptor (Mantuano et al. 2020). This caused neurite outgrowth in a neuronal cell line and migration of Schwann cells, thus indicating relevance in the central and peripheral nervous system, respectively. Another report showed that recPrP induced phosphorylation of Erk1/2 and additional signalling causing neuronal differentiation of stem cells (Martellucci et al. 2019a, b). As in other conditions mentioned earlier (Amin et al. 2016), this required expression of PrP at the cell surface suggesting an underlying homophilic encounter between sPrP as ligand and PrP as (co-)receptor. In sum, interaction of PrP with diverse partners is key to its established roles in regulating (stem) cell proliferation, maintenance, and viability as well as morphological and functional differentiation (such as epithelial-to-mesenchymal transition) in the central nervous system and beyond (Brethour et al. 2017; Lee and Baskakov 2013; Mehrabian et al. 2015; Prodromidou et al. 2014; Steele et al. 2006; Zhang et al. 2006). These fine-tuned processes are relevant during development and for regenerative processes yet may be corrupted in pathogenic conditions such as cancer. The role played by sPrP in these regards is out for debate and investigation.

Although it seems at least possible, if not likely, that sPrP is the physiological correlate in many of the pathophysiological processes covered in this review, detailed studies on this are lacking to date. All of the aforementioned studies using recPrP or transgenically expressed anchorless PrP as a ‘proxy’ for physiological sPrP are -without doubt- very interesting and may help to unravel sPrP’s real functions. However, pending further direct proof and considering the possibly relevant structural differences of these forms compared to physiologically shed PrP (Fig. 3), one should, at least at the present state, be careful with generalizing experimental findings. In this consideration, we would rather disagree with recent statements that these PrP versions are ‘basically the same as’ shed PrP (Dexter and Kong 2021b).

Apart from the focus on released PrP fragments, proteolytic processing may also have implications from the perspective of membrane-bound PrP. In many of the biological implications discussed herein, a gradual engagement of proteolytic cleavages is conceivable: by releasing the N-terminal half of PrP, α-cleavage may inhibit certain interactions, while simultaneous production of the C1 fragment with its exposed hydrophobic sequence likely enables others. In this scenario, the PrP shedding by ADAM10 might be a mechanism to regulate or terminate all PrP interactions and downstream effects. This assumption could be particularly important for two partially connected aspects, namely the established involvement of cell surface PrP in both, diverse cellular signalling cascades (Chiarini et al. 2002; Mattei et al. 2020; Mouillet-Richard et al. 2000) and cell adhesion (Kaiser et al. 2012; Málaga-Trillo et al. 2009; Mangé et al. 2002; Petit et al. 2013; Solis et al. 2013). Moreover, PrP and its C1 fragment are highly enriched on EVs and may serve important regulatory functions regarding the fate of EVs upon interaction with recipient cells and delivery of cargo and information (Brenna et al. 2020; D’Arrigo et al. 2021; Falker et al. 2016; Guo et al. 2015; Linsenmeier et al. 2018; Vella et al. 2008). EVs are considered as rather stable extracellular structures able to cross tissue borders, including the blood–brain barrier, whereas half-life of sPrP as a soluble factor in tissue environment or body fluids might be rather short. Strikingly, EVs also carry the active form of ADAM10 and, though not reported yet, it is conceivable that PrP shedding continues on EVs, as has been shown for other ADAM10 substrates (Folkesson et al. 2015; Padro et al. 2013; Pérez-González et al. 2020; Stoeck et al. 2006). It appears tempting to speculate (and investigate) whether EVs could function as a ‘carrier rocket’ enabling transport to distant target organs, followed by subsequent local release of sPrP to exhibit its functions.

## Conclusion and outlook

**Fig. 4:**
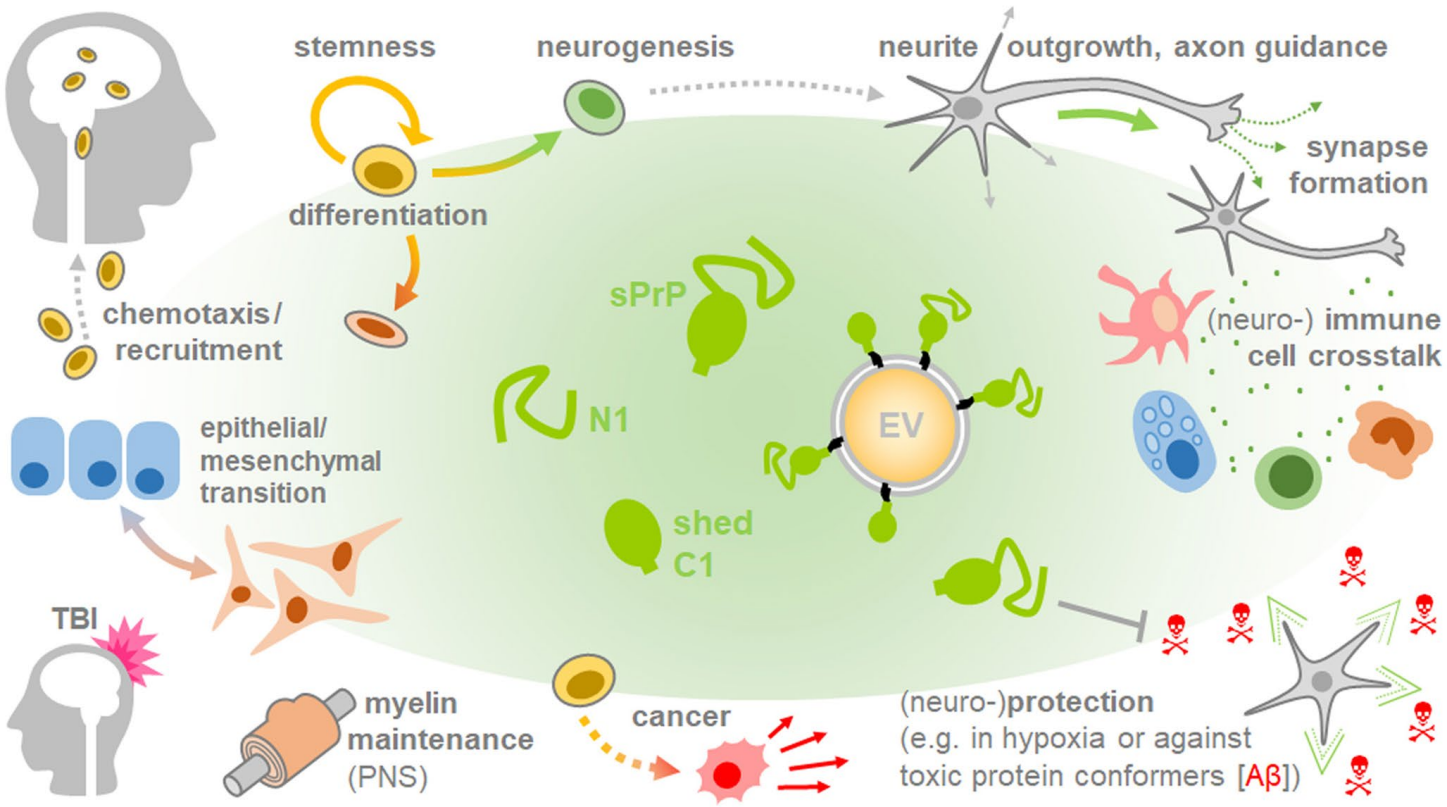
Overview of potential (patho)physiological implications of sPrP. A plethora of studies suggest that released fragments of PrP are linked with intrinsic functions, which may partially explain the multitude of roles attributed to this evolutionary conserved protein. Putative roles of sPrP addressed in this review may be beneficial (e.g., in neurodegeneration, development/differentiation) or detrimental (e.g., in cancer progression or immune response aggravation). It should be noted, however, that many of the roles discussed herein are, thus far, rather speculative and based on the combination of described PrP participation on the one hand, and documented involvement of (increased) ADAM10 expression/activity on the other hand. Clear experimental proof in these and other conditions is mostly pending and will eventually require use of research tools able to discriminate between different PrP derivatives

As it stands now, clear conclusions on whether PrP shedding and sPrP play harmful (e.g., in cancer) or beneficial roles (e.g., in neurodegeneration) cannot be easily drawn — it all rather seems to be a matter of perspective (and pathophysiological context). Plenty of biological implications may arise for the ADAM10-mediated shedding and its product sPrP (summarized in Fig. 4). And even though shedding may not be of mechanistic relevance in certain processes mentioned herein, considering the broad expression pattern of both, PrP and its sheddase ADAM10, alterations in sPrP levels in body fluids may qualify as diagnostic tool in some pathological conditions. Also, PrP shedding may well affect processes in organs/tissues not covered in this review. However, all of this requires detailed analyses and new research tools able to reliably differentiate between sPrP and other PrP derivatives. While sPrP-specific antibodies for murine models are available, identification of the shedding site and generation of respective antibodies for the human system and other species will fuel further studies and provide insight into both, biological effects and biomarker potential of sPrP. Moreover, given that PrP appears to be exclusively shed by ADAM10 (with no contribution by other proteases, such as the closely related and often redundantly working ADAM17), detection of sPrP as a surrogate read-out could become a convenient way to investigate the efficacy of any pharmacological approaches aiming to manipulate ADAM10 activity in general. With regard to PrP, we recently presented a substrate-specific approach that, depending on the applied PrP-directed ligand, enables stimulated shedding as well as (transient) downregulation of total PrP (Linsenmeier et al. 2021). Future studies into this direction, but also on endogenous regulators (e.g., certain tetraspanin molecules (Matthews et al. 2017; Saint-Pol et al. 2017; Seipold et al. 2018)) or pharmacological modulators of ADAM10 trafficking, maturation and activity, could pave the way for future therapeutic avenues in neurodegeneration and beyond.
